# Prognostic value of inflammation-based scores in patients with osteosarcoma

**DOI:** 10.1038/srep39862

**Published:** 2016-12-23

**Authors:** Bangjian Liu, Yujing Huang, Yuanjue Sun, Jianjun Zhang, Yang Yao, Zan Shen, Dongxi Xiang, Aina He

**Affiliations:** 1 Department of Neurology, Affiliated Sixth People’s Hospital, Shanghai Jiaotong University, No. 600, Yishan Road, 200233 Shanghai, People’s Republic of China; 2 Department of Oncology, Affiliated Sixth People’s Hospital, Shanghai Jiaotong University, No. 600, Yishan Road, 200233 Shanghai, People’s Republic of China; 3 Department of Medicine, Brigham and Women’s Hospital and Harvard Medical School, Boston, MA 02115, USA

## Abstract

Systemic inflammation responses have been associated with cancer development and progression. C-reactive protein (CRP), Glasgow prognostic score (GPS), neutrophil-lymphocyte ratio (NLR), platelet-lymphocyte ratio (PLR), lymphocyte-monocyte ratio (LMR), and neutrophil-platelet score (NPS) have been shown to be independent risk factors in various types of malignant tumors. This retrospective analysis of 162 osteosarcoma cases was performed to estimate their predictive value of survival in osteosarcoma. All statistical analyses were performed by SPSS statistical software. Receiver operating characteristic (ROC) analysis was generated to set optimal thresholds; area under the curve (AUC) was used to show the discriminatory abilities of inflammation-based scores; Kaplan-Meier analysis was performed to plot the survival curve; cox regression models were employed to determine the independent prognostic factors. The optimal cut-off points of NLR, PLR, and LMR were 2.57, 123.5 and 4.73, respectively. GPS and NLR had a markedly larger AUC than CRP, PLR and LMR. High levels of CRP, GPS, NLR, PLR, and low level of LMR were significantly associated with adverse prognosis (P < 0.05). Multivariate Cox regression analyses revealed that GPS, NLR, and occurrence of metastasis were top risk factors associated with death of osteosarcoma patients.

Osteosarcoma is the most common primary tumor of bone, predominantly affecting adolescents and young adults^1^,^2^. In the past, when surgery was the only therapy, most patients died within one year following diagnosis, and the overall 5-year survival rate was around 10%^3^. The introduction of multi-disciplinary treatment led to 5-year survival rate of approximately 70%^1^,^3^. The established prognostic factor were Enneking surgical criteria^4^, tumor site^5^, alkaline phosphatase^6^, lactate dehydrogenase^7^, and etc. However, big variations in clinical outcomes were observed with these prognostic factors. For instance, heterogeneous prognoses were frequently found in the patients with the same stage. Identification of novel prognostic factors will help us distinguish high-risk patients who need specific therapy, and may lead to more effective therapies to improve clinical outcomes. Although many new factors, such as MicroRNA-191^8^, survivin^9^, long non-coding RNA HOTTIP^10^, have revealed their prognostic significance in osteosarcoma, their detections were costly and inconvenient. Therefore, identification of easily-assessed factors that can predict outcome of osteosarcoma more precisely is required.

Cancer-related inflammation has been identified as the seventh hallmarks of cancer^11^, in addition to self-sufficiency in growth signals, insensitivity to growth-inhibitory signals, evasion of apoptosis, limitless replicative potential, sustained angiogenesis, and tissue invasion and metastasis^12^. Inflammatory microenvironment promotes the development of tumors via promoting angiogenesis and metastasis, subverting adaptive immune responses, and altering responses to hormones and chemotherapeutic agents^13^. The addition of anti-inflammatory drugs during chemotherapy has been suggested to be a new effective treatment to increase patient survival^14^. Due to the association of inflammation in cancer development, the prognostic significance of several inflammation biomarkers and hematological indices, including the C-reactive protein (CRP), Glasgow prognostic score (GPS), neutrophil-lymphocyte ratio (NLR), platelet-lymphocyte ratio (PLR), lymphocyte-monocyte ratio (LMR), and neutrophil-platelet score (NPS) have been evaluated in various malignancies. CRP, GPS, PLR and NLR were reported to be significantly associated with both overall survival and disease-free survival of patients with gastric cancer^15^. In metastatic colorectal cancer, high NLR, PLR, and low LMR were significantly linked to decreased survival time^16^. Elevated GPS, NLR, and PLR were also reported to be associated with poor survival of hepatocellular carcinoma^17^. High NPS level was significantly associated with poor survival in a variety of common cancers^18^. However, little was known about the prognostic role of the inflammation biomarkers in osteosarcoma.

In this retrospective study, we evaluated the clinical significance of pre-treatment inflammation-based scores and determined the independent prognostic factors for patients with osteosarcoma.

## Results

### Patient characteristics

**Figure 1:**
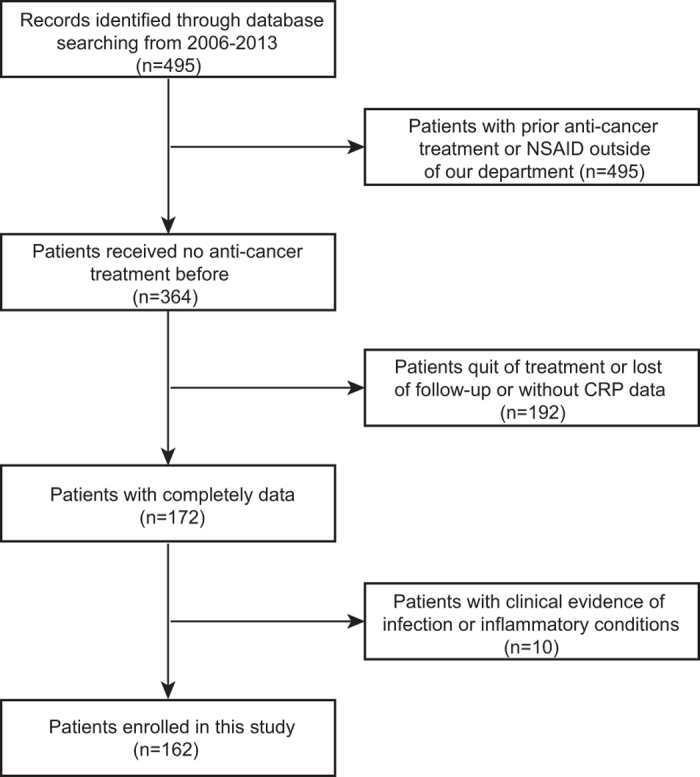
Flow chart for patients’ selection in this study.

This study consisted of 162 osteosarcoma patients with complete clinical data (Fig. 1) including 96 male and 66 female patients. Patients with large tumor may have cancer fever without evidence of infection. Twenty-one patients with cancer fever were excluded in this study, they have already accepted NSAID or anti-cancer treatment outside of our department. No studies have been reported that cancer fever could affect the blood routine test. In this case, three patients with cancer fever were enrolled and they were indispensable among our patient population. Table 1 lists the main features of the analyzed patients. The median age was 18 year-old, and the majority of tumors (89.5%) located in extremities. A total of 93.8% patients had a KPS of ≥80, and 80.9% patients received neoadjuvant chemotherapy. 143 patients were diagnosed as stage I–II according to Enneking surgical staging criteria, and the rest were stage III. The number of patients suffered from pathological fracture, local recurrence and distant metastasis were 18, 31 and 78, respectively. All enrolled patients underwent surgery followed by 8–14 cycles of adjuvant chemotherapy.

### The optimal cut-off value for inflammation-based scores

ROCs were performed, and the optimal threshold of inflammation-based score was obtained when the Youden index was maximal. The optimal cut-off points of NLR, PLR, and LMR were 2.57 (Youden index, 0.326), 123.5 (Youden index, 0.200) and 4.73 (Youden index, 0.199), respectively. The cut-off levels of GPS and CRP were described in Data collection of the Methods section. Patients were divided into two groups based on these cut-off values, with either low or high value. Of the 162 patients, 126 (77.8%) had a GPS of 0, while 31 (19.1%) and 5 (3.1%) patients showed a GPS of 1 and 2, respectively. The patient numbers of NPS of 0, 1, and 2 were 152 (93.8%), 9 (5.6%), and 1 (0.6%), respectively. The patient numbers of high groups of CRP, NLR, PLR, and LMR were 25 (15.4%), 53 (32.7%), 100 (61.7%), and 65 (40.1%), respectively.

### Survival analysis

**Figure 2:**
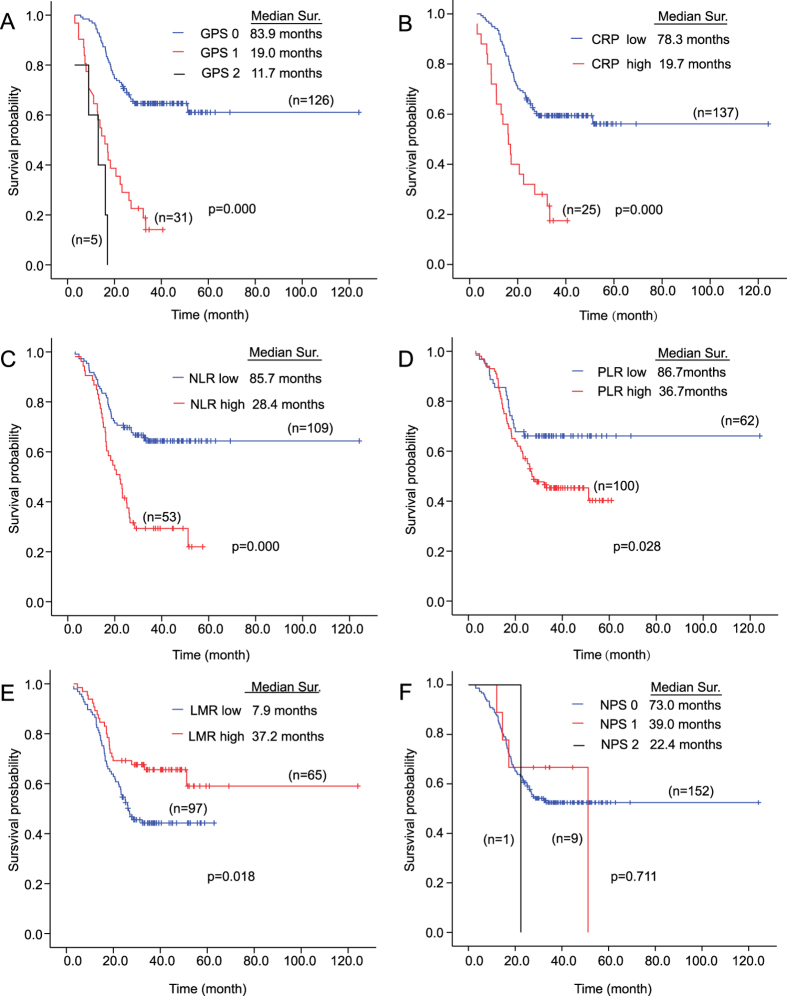
Kaplan Meier survival curves for overall survival according to inflammation-based scores in 162 osteosarcoma patients. (**A**) Glasgow Prognostic Score; (**B**) The C-reactive protein; (**C**) Neutrophil-lymphocyte ratio; (**D**) Platelet-lymphocyte ratio; (**E**) Lymphocyte-monocyte ratio; (**F**) Neutrophil-platelet score.

The median OS of all patients was 28.2 (range 3.1–124.1) months. Eighty-six (53.1%) patients were alive at the end of the follow-up period (Fig. 2). Among 76 patients died at the end of the follow-up period, the number of patients died due to infection of peripherally inserted central catheter (PICC), bone marrow depression by chemotherapy, and respiratory failure induced by pneumothorax and hydrothorax were 1, 1, and 74, respectively (Table 2). The survival curve revealed that the median OS of patients with GPS of 0 was 83.9 months, while only 19.0 and 11.7 months in score 1 and 2 groups respectively (P = 0.000) (Fig. 2A). Patients with high CRP had significant shorter OS than patients with low CRP (median OS, 19.7 vs 78.3 months, P = 0.000) (Fig. 2B). Similar results were observed in NLR (median OS, 28.4 vs 85.7 months, P = 0.000) (Fig. 2C) and PLR (median OS, 36.7 vs 86.7 months, P = 0.028) (Fig. 2D). Low LMR group, rather than high LMR, was associated with worse survival (median OS, 37.2 vs 7.9 months, P = 0.018) (Fig. 2E). The median OS of patients with NPS of 0, 1, and 2 was 73.0, 39.0, and 22.4 months, respectively (P = 0.711) (Fig. 2F). Taken together, all inflammation-based scores, except NPS, were correlated with overall survival.

The univariate and multivariate analysis results were shown in Table 3. The univariate analysis indicated that tumor site (P = 0.022), Enneking’s surgical staging (P = 0.000), occurrence of metastasis (P = 0.000), GPS (P = 0.000), CRP (P = 0.000), NLR (P = 0.000), PLR (P = 0.030) and LMR (P = 0.020) were associated with OS. Multivariate analysis for factors above demonstrated that occurrence of metastasis (HR, 10.407; 95% CI, 5.265–20.570; P = 0.000), high level of NLR (HR, 2.097; 95% CI, 1.202–3.658; P = 0.009), and high level of GPS (HR, 2.250; 95% CI, 1.222–4.145; P = 0.009) were independent unfavorable prognostic factors.

As shown in Table 4, for histological subtypes, there were 148, 6, 6 and 2 patients diagnosed with conventional, telangiectatic, intramedullary and periosteal osteosarcoma, respectively. No obvious relations were observed between these histological subtypes and either GPS or NLR in other tumors, including gastric or pancreatic cancers^19^,^20^; the number of non-conventional osteosarcoma was too small in our current study so that no further statistical analysis could be performed.

### Discriminatory ability of inflammation-based scores

**Figure 3:**
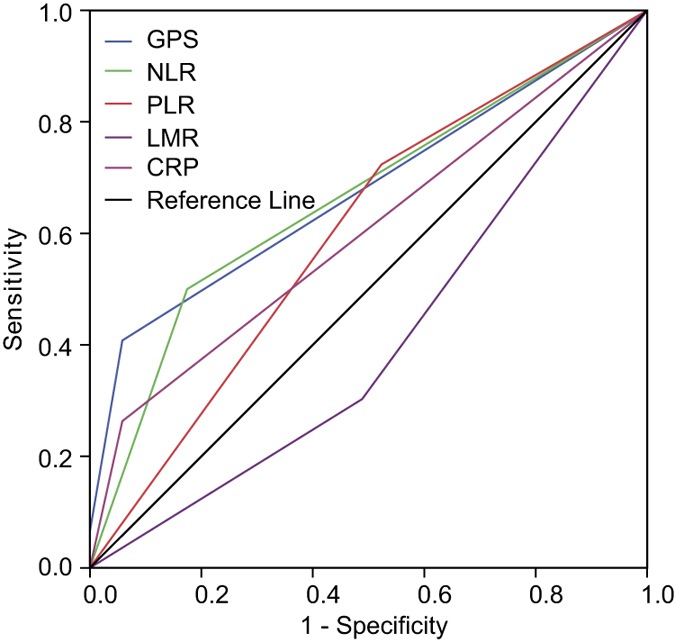
Comparison of the area under the ROC curve of inflammation-based scores to predict overall survival. ROC = receiver operating characteristic; GPS = Glasgow prognostic score; CRP = the C-reactive protein; NLR = eutrophil-lymphocyte ratio; PLR = platelet-lymphocyte ratio; LMR = lymphocyte-monocyte ratio; NPS = neutrophil-platelet score.

We compared the significance of GPS, CRP, NLR, PLR and LMR to discriminate between survivors and non-survivors using ROC curves. As shown in Table 5, the AUC of GPS was 0.677 (95% CI, 0.592–0.761), with a sensitivity of 40.8% and a specificity of 94.2%. The AUC of NPS was 0.504 (95% CI, 0.415–0.594), with a sensitivity of 6.6% and a specificity of 94.2%. The AUC of CRP was 0.603 (95% CI, 0.514–0.691), with a sensitivity of 26.3% and a specificity of 94.2%. The AUC of NLR was 0.663 (95% CI, 0.578–0.748), with a sensitivity of 50.0% and a specificity of 82.6%. The AUC of PLR was 0.600 (95% CI, 0.513–0.687), with a sensitivity of 72.4% and a specificity of 47.7%. The AUC of LMR was 0.407 (95% CI, 0.320–0.495), with a sensitivity of 30.3% and a specificity of 51.2%. Notably, GPS and NLR showed higher AUC values than CRP, PLR, LMR, and NPS (Table 5 and Fig. 3).

## Discussion

The accuracy of prognostic evaluation is critical for the medical care of patients with osteosarcoma. Adverse predictive factors, including detectable primary metastases, axial or proximal extremity tumor site, large tumor size, and older age, were well established^21^. In addition to these tumor characteristics, systemic inflammation also affected the prognosis of osteosarcoma. Elevated CRP was reported to be significantly associated with poor prognosis^22^,^23^,^24^,^25^. Pretreatment neutrophils count is an independent prognostic factor of extremity osteosarcoma. Moreover, low level of LMR was regarded to be related with adverse OS in osteosarcoma by Liu T, *et al*.^26^. However, the clinical prognostic value of GPS, PLR, NLR, and NPS in osteosarcoma remained poorly defined.

Elevated CRP reflected increased systemic inflammatory response, which involved in tumor development and progression. GPS is a prognostic score comprised of serum CRP and albumin levels. It was first introduced by Forrest *et al*., who evaluated its prognostic significance in inoperable non-small-cell lung cancer^27^. After that, GPS was found to be a prognostic factor in various other tumors, such as renal clear cell cancer, gastric cancer, gallbladder cancer, and Esophageal squamous cell carcinoma^15^,^28^,^29^,^30^. Patients with GPS more than zero have elevated CRP or hypoalbuminaemia or even both of them, implying GPS presents not only inflammation status but also nutritional status of cancer patients. Therefore, we speculate that GPS might be a better predictor of the prognosis of cancer than CRP. Patients with high GPS in our study accepted the same therapy with the rest patients. Our results indicated that GPS, rather than CRP, was an independently prognostic factor of osteosarcoma (Table 3). Moreover, GPS also showed a higher sensitivity of 40.8% in comparison with the sensitivity of 26.3% of CRP, while their specificities were the same (Table 5).

Cancer-related inflammation has a role in cancer development and progression^31^,^32^. Neutrophilia, thrombocytosis, monocytosis and lymphopenia tend to represent a nonspecific response to cancer-related inflammation and are associated with poor survival in cancers^32^,^33^,^34^,^35^,^36^. Neutrophils interact with tumor cells by producing cytokines and chemokines, which affects tumor cells’ proliferation, angiogenesis, and metastases^37^. Tumor-associated macrophages, which arise from blood monocytes, promoted tumor progression and metastases^38^. Lymphocytes play a major role in the immune response by mediating the immunologic destruction of various cancers^39^,^40^. Platelets were also reported to act as chemoattractants, increasing the migration of ovarian cancer cells^41^. NLR, PLR, LMR, and NPS have been shown to be independent risk factors in various malignant tumors^18^,^42^,^43^,^44^. These factors when combined may have stronger prognosis value than any single one. Moreover, LMR was suggested as an independent prognostic factor for OS in patients with osteosarcoma by Liu T, *et al*.^26^. However, among these prognostic scores, only NLR was an independent risk factor in osteosarcoma in our research, in which patients with high NLR accepted the same therapy with patients with the rest patients (Table 3). The difference might be caused by heterogeneity in patient populations between these two studies. For instance, Liu T, *et al*. enrolled patients with no prior pre-operative anticancer treatment, but as high as 80.9% patients received neoadjuvant chemotherapy in our study.

Using ROC curve analysis, we have shown that optimal cut-off points of NLR, PLR, and LMR were 2.57, 123.5 and 4.73, respectively. Optimal thresholds of NLR, PLR and LMR in our study were similar to the studies in other malignants^42^,^43^,^44^. The AUC of GPS, CRP, NLR, PLR, LMR, and NPS were compared, and GPS and NLR had a markedly larger AUC than CRP, PLR, LMR, and NPS (Table 5 and Fig. 3), which was consistent with the results of multivariate analysis (Table 3).

It is worth noting that our study is the first attempt to evaluate the prognosis significance of these inflammation-based prognostic scores in patients with osteosarcoma. In addition, inflammation-based scores are simple and comprised of components of blood test with low cost. The establishment of predictive model based on inflammation is of great value for the patients in developing countries. However, there are still several potential limitations in the present study. First and most importantly, this is a retrospective, single-institution, and small-sample-size study, providing a lower level of confidence than those randomized controlled trials. Second, the sample size of 162 patients enrolled in our study was not enough to separate into two groups to derive the parameters and confirm their utility, which might cause overestimation of results. Third, the age distribution is atypical in our population, as most of osteosarcoma occurs at pediatric age. Fourth, blood samples were not obtained at same time and with no repeated test, which could introduce irreconcilable bias and negate the utility of the test, because blood parameters are dynamic. Finally, the heterogeneity in the treatments of patients might also affect the results. Our results should be interpreted with cautious according to defections above.

In conclusion, the present study indicated that abnormal pretreatment inflammation-based prognostic scores, such as GPS > 0, CRP > 10 mg/L, NLR > 2.57, PLR > 123.5, and LMR ≤ 4.73, were inversely associated with OS in osteosarcoma. Moreover, occurrence of metastasis, GPS and NLR are robust predictors of osteosarcoma survival. Patients with these two risk factors may need more aggressive chemotherapy and closely follow-up to improve clinical outcomes, according to our observations. Due to the limitations of retrospective studies, further prospective studies are still warranted.

## Methods

### Patients

We retrospectively reviewed the electronic medical records for 495 patients with osteosarcoma who were accepted by our department from January 2006 to December 2013. The inclusion criteria for primary studies were as follows: (i) All patients diagnosed with histologically confirmed osteosarcoma. (ii) All patients received no anti-cancer treatment before. Patients with either of the following diagnoses were excluded from the final analysis, including patients who already have neutrophilia, high CRP, or high procalcitonin, or if they have clinical evidence of infection or any other inflammatory conditions, or patients who were treated with anti-cancer therapy or non-steroid anti-inflammatory drug (NSAID) previously, or those whose clinical data were incomplete. Antibiotics were not prescribed for patients enrolled. NSAID is an efficient approach for cancer fever treatment, which is also wildly used in patients with osteosarcoma^45^,^46^,^47^. NSAID was reported to affect blood test^48^,^49^, then patients with record of NSAID treatment before blood test were excluded. The study was approved by the medical ethics committee of Shanghai Jiaotong University Affiliated Sixth People’s Hospital.

### Data collection

Clinical data including sex, age, Enneking stage, Karnofsky performance status (KPS) score, tumor location, neoadjuvant chemotherapy, histologic type, pathological fracture, local recurrence, and occurrence of metastasis were collected. Routine laboratory measurements were performed before any anti-cancer treatments. Data including CRP, albumin, neutrophil count, lymphocyte count, and platelet count were used to calculate GPS, NLR, PLR, LMR, and NPS.

The GPS was constructed as following^27^: patients with both elevated CRP(>10 mg/L)^22^ and hypoalbuminaemia (<35 g/L) were allocated a score of 2; patients with neither of the biochemical abnormalities were allocated a score of 0; and others with either of the biochemical abnormalities were allocated a score of 1. The NPS was calculated as follows^18^: patients with a neutrophil count ≤7.5 × 10^9^/L and platelets ≤400 × 10^9^/L were scored 0, patients with neutrophils >7.5 × 10^9^/L or platelets >400 × 10^9^/L were scored 1 and patients with both neutrophils >7.5 × 10^9^/L and platelets >400 × 10^9^/L were scored 2. NLR was defined as a simple ratio of the absolute neutrophil count over lymphocyte count. PLR was defined as a ratio of the platelet count over the absolute lymphocyte count. LMR was defined as a ratio of the absolute lymphocyte count over the monocyte count.

### Patient follow-up

All 162 patients were followed after the completion of adjuvant chemotherapy. The intervals for follow-up were every 3 months for the first 4 years, then every 6 months until December 10, 2015. The routine follow-up examinations included physical examination, laboratory tests, chest CT and radiographs of the operated limb. Bone scans were performed every 6 months. The study was approved by the medical ethics committee of Shanghai Jiaotong University Affiliated Sixth People’s Hospital.

### Statistical analysis

All statistical analyses were performed by SPSS statistical software (Version 17.0, IBM Corp.). Overall survival (OS) was defined as the time from the date of diagnosis to the date of the last follow-up or death from any cause. The optimal cut-off values of NLR, PLR and LMR were determined by receiver operating characteristic (ROC) analysis, using OS as end-point. Kaplan-Meier analysis was performed to plot the survival curve. To determine the independent prognostic factors, univariate analysis and multivariate analysis, expressed as hazard ratios (HR) and 95% confidence, were performed by Cox Regression Model. Area under the curve (AUC) of ROC was calculated and compared to evaluate the discriminatory ability of the inflammation-based prognostic scores. P values were two-sided, and P < 0.05 was considered statistically significant.

## Additional Information

**How to cite this article:** Liu, B. *et al*. Prognostic value of inflammation-based scores in patients with osteosarcoma. *Sci. Rep.* **6**, 39862; doi: 10.1038/srep39862 (2016).

**Publisher's note:** Springer Nature remains neutral with regard to jurisdictional claims in published maps and institutional affiliations.

## Figures and Tables

**Table 1:** Patient characteristics.

Characteristic	
Gender	
Female	66 (40.7)
Male	96 (59.3)
Age/year	
<18	73 (45.1)
≥18	89 (54.9)
Tumor site	
Extremities	145 (89.5)
Non-extremities	17 (10.5)
Enneking’s surgical staging	
I/II	143 (88.3)
III	19 (11.7)
Karnofsky performance status score	
≥80	152 (93.8)
≤70	10 (6.2)
Neoadjuvant chemotherapy	
Yes	131 (80.9)
No	31 (19.1)
Pathological fracture	
Yes	18 (11.1)
No	144 (88.9)
Local recurrence	
Yes	31 (19.1)
No	131 (80.9)
Metastasis	
Yes	78 (48.1)
No	84 (51.9)

Data presented are number (%).

**Table 2:** Methods of therapy and causes of death for the osteosarcoma patients with high GPS and NLR.

Factors	NLR			GPS		
	0	1	P	0	1/2	P
Methods of therapy						
Neoadjuvant chemotherapy			0.101			0.310
No	17	14		22	9	
Yes	92	39		104	27	
Operation			0.535			0.843
Salvage	85	39		96	28	
Amputation	24	14		30	8	
Cause of death (N = 76)			—			—
Infection	1	0		0	1	
Bone marrow depression	0	1		1	0	
Respiratory failure	37	37		44	30	

GPS = Glasgow prognostic score; NLR = neutrophil-lymphocyte ratio.

**Table 3:** Univariate and multivariate Cox proportional hazard regression analyses of overall survival.

Factor	Univariate analysis		Multivariate analysis	
	HR(95%CI)	P	HR(95%CI)	P
Gender		0.417		
Female	Reference			
Male	1.213 (0.761–1.934)			
Age/year		0.479		
<18	Reference			
≥18	0.850 (0.542–1.333)			
Tumor site		0.022		0.061
Extremities	Reference		Reference	
Non-extremities	2.058 (1.110–3.814)		1.907 (0.971–3.746)	
Enneking’s surgical staging		0.000		0.509
I/II	Reference		Reference	
III	2.751 (1.559–4.855)		1.228 (0.668–2.255)	
Karnofsky performance status score		0.262		
≥80	Reference			
≤70	1.561 (0.717–3.399)			
Neoadjuvant chemotherapy		0.311		
No	Reference			
Yes	0.756 (0.441–1.298)			
Pathological fracture		0.258		
No	Reference			
Yes	0.618 (0.268–1.423)			
Local recurrence		0.162		
No	Reference			
Yes	1.449 (0.861–2.439)			
Metastasis		0.000		0.000
No	Reference		Reference	
Yes	12.751 (6.642–24.479)		10.407 (5.265–20.570)	
NLR		0.000		0.009
0	Reference		Reference	
1	2.645 (1.682–4.160)		2.097 (1.202–3.658)	
PLR		0.030		0.186
0	Reference		Reference	
1	1.746 (1.056–2.887)		0.676 (0.379–1.207)	
LMR		0.020		0.796
0	Reference		Reference	
1	0.559 (0.342–0.912)		0.927 (0.524–1.641)	
GPS		0.000		0.009
0	Reference		Reference	
1/2	5.596 (2.501–5.170)		2.250 (1.222–4.145)	
CRP		0.000		0.709
0	Reference		Reference	
1	3.133 (1.874–5.239)		1.166 (0.521–2.610)	
NPS		0.803		
0	Reference			
1/2	1.101 (0.515–2.358)			

GPS = Glasgow prognostic score; CRP = the C-reactive protein; NLR = neutrophil-lymphocyte ratio; PLR = platelet-lymphocyte ratio; LMR = lymphocyte-monocyte ratio; NPS = neutrophil platelet score; HR = hazard ratios; CI = confidence interval.

**Table 4:** The association between histological subtypes and GPS, NLR and occurrence of metastasis.

Factor	Histological subtypes			
	Conventional	Telangiectatic	Intramedullary	Periosteal
Metastasis				
No	75	3	4	2
Yes	73	3	2	0
NLR				
0	101	3	3	2
1	47	3	3	0
GPS				
0	117	4	3	2
1/2	31	2	3	0

Data presented are number. GPS = Glasgow prognostic score; NLR = neutrophil-lymphocyte ratio.

**Table 5:** Sensitivity, specificity, positive likelihood ratio, and negative likelihood ratio for inflammation-based scores.

Inflammation-based scores	Cut-off value	AUC	Sensitivity (%)	Specificity (%)	Positive likelihood ratio	Negative likelihood ratio
GPS	Unavailable*	0.677	40.8	94.2	7.034	0.628
CRP	10 mg/L**	0.603	26.3	94.2	4.534	0.782
NLR	2.57	0.663	50.0	82.6	2.874	0.605
PLR	123.50	0.600	72.4	47.7	1.384	0.579
LMR	4.73	0.407	30.3	51.2	0.621	1.361
NPS	Unavailable*	0.504	6.6	94.2	1.138	0.992

GPS = Glasgow prognostic score; CRP = the C-reactive protein; NLR = neutrophil-lymphocyte ratio; PLR = platelet-lymphocyte ratio; LMR = lymphocyte-monocyte ratio; NPS = neutrophil platelet score; ROC = eceiver operating characteristic analysis; AUC = the area under the curve.

^*^GPS and NPS are categorical variables.

^**^The cut-off value of CRP was determined by reference rather than ROC curve.
